# Genetic Compatibility and Virulence of Reassortants Derived from Contemporary Avian H5N1 and Human H3N2 Influenza A Viruses

**DOI:** 10.1371/journal.ppat.1000072

**Published:** 2008-05-23

**Authors:** Li-Mei Chen, C. Todd Davis, Hong Zhou, Nancy J. Cox, Ruben O. Donis

**Affiliations:** Influenza Division, National Center for Immunization and Respiratory Diseases, Centers for Disease Control and Prevention, Atlanta, Georgia, United States of America; Mount Sinai School of Medicine, United States of America

## Abstract

The segmented structure of the influenza virus genome plays a pivotal role in its adaptation to new hosts and the emergence of pandemics. Despite concerns about the pandemic threat posed by highly pathogenic avian influenza H5N1 viruses, little is known about the biological properties of H5N1 viruses that may emerge following reassortment with contemporary human influenza viruses. In this study, we used reverse genetics to generate the 63 possible virus reassortants derived from H5N1 and H3N2 viruses, containing the H5N1 surface protein genes, and analyzed their viability, replication efficiency, and mouse virulence. Specific constellations of avian–human viral genes proved deleterious for viral replication in cell culture, possibly due to disruption of molecular interaction networks. In particular, striking phenotypes were noted with heterologous polymerase subunits, as well as NP and M, or NS. However, nearly one-half of the reassortants replicated with high efficiency *in vitro*, revealing a high degree of compatibility between avian and human virus genes. Thirteen reassortants displayed virulent phenotypes in mice and may pose the greatest threat for mammalian hosts. Interestingly, one of the most pathogenic reassortants contained avian PB1, resembling the 1957 and 1968 pandemic viruses. Our results reveal the broad spectrum of phenotypes associated with H5N1/H3N2 reassortment and a possible role for the avian PB1 in the emergence of pandemic influenza. These observations have important implications for risk assessment of H5N1 reassortant viruses detected in surveillance programs.

## Author Summary

The influenza pandemics of 1957 and 1968 were caused by hybrid viruses consisting of a mixture of human and avian influenza genes. The introduction of avian genes resulted in a sudden change of the virus surface antigens, allowing its worldwide spread due to lack of immunity in the population. The highly pathogenic avian influenza H5N1 virus has continued its spread in domestic and wild birds in Asia, Europe, and Africa. Although H5N1 infection in humans is rare and person-to-person transmission is very inefficient, the steady accumulation of human cases has raised concern over the possible reassortment between H5N1 and human seasonal influenza resulting in a virus with new surface antigens and pandemic potential. In this study, we used recombinant DNA technology to generate a systematic collection of hybrid viruses (with genes from human and avian viruses) bearing H5N1 surface antigens and analyzed their properties in cell culture and in mice. The H5N1 hybrid viruses revealed a broad range of viability and multiplication capacity in cell cultures. In addition, several H5N1 hybrid viruses were highly virulent in mice. Results from this systematic analysis provide important insight to support risk assessment of reassortant H5N1 avian influenza viruses.

## Introduction

The emergence of an influenza virus that will cause a pandemic is inevitable and therefore preparedness is mandatory. The new pandemic influenza virus is likely to carry a hemagglutinin (HA) gene other than the currently circulating H1 and H3 lineages in order to escape immunity in the human population. However, we cannot predict the mechanism by which the pandemic influenza virus will emerge. One possibility is the transfer of an avian influenza virus from birds to humans, made possible by adaptive mutations, as postulated for the 1918 pandemic [1],[2]. Another possible scenario would follow the paradigm of the H2N2 and H3N2 influenza pandemics of 1957 and 1968 in which avian virus genes were incorporated into circulating human influenza viruses by reassortment [3], giving rise to viruses with novel surface antigens; i.e. antigenic shift. The segmented structure of the viral genome facilitates exchange of gene segments between two viruses co-infecting a single host cell. Dual infection with avian and human influenza viruses and subsequent reassortment may occur in hosts that are susceptible to both kinds of viruses and serve as mixing vessels that generate novel reassortants [4],[5].

Wild aquatic birds are the natural reservoirs for influenza A viruses and have been found to harbor each of the 16 known HA subtypes [6]. Highly pathogenic avian influenza (HPAI) H5N1 viruses are now enzootic among wild birds and poultry in three continents (http://www.who.int). Since 1997, when HPAI H5N1 viruses first emerged in Hong Kong to cause human respiratory illness and death, over 360 laboratory-confirmed human infections have been reported. Most human infections are caused by contact with infected poultry and to date H5N1 viruses have not yet acquired the ability to transmit efficiently among humans.

A major obstacle to transmission of the H5N1 virus among humans is thought to be the preferred receptor specificity of the H5 HA towards sialic acid (SA) with α2,3 linkage to galactose (the so-called avian receptor) [7],[8]. A switch of receptor specificity towards α2,6 linked SA (the human receptor) is considered to be a pre-requisite for sustained human to human transmission [9],[10]. However, it is not known whether other genes from H5N1 viruses would confer virulence and transmissibility in humans. It has been shown that a reassortant virus with the HA and NA from an H3N2 human virus and the PB2, PB1, PA, NP, M, and NS (so-called internal genes) of an H5N1 virus did not transmit efficiently in a ferret model [11]. (In this report, the term “internal genes” refers to the gene constellation comprising PB2, PB1, PA, NP, M, and NS, although the M gene encodes for the M2 protein, which is surface-exposed in virions.) The internal genes from this avian H5N1 virus were therefore postulated to lack at least one essential functional attribute to initiate a human pandemic. These critical function(s) might be acquired through a reassortment event between the H5N1 virus with a circulating human H3N2 influenza virus that generates the appropriate gene constellation.

In theory, a single reassortment event between two influenza A viruses can yield up to 254 (2^8^ minus two parental viruses) hybrid genotypes. However, the few available reports suggest that the number of natural or experimental reassortants is likely to be smaller [5],[12],[13],[14],[15],[16]. Reliable estimates of the expected frequency of hybrid genotypes resulting from dual infections are not possible in the absence of systematic studies on human-avian influenza reassortment. Comprehensive *in vivo* co-infection studies and *in vitro* evaluations of all the reassortant genotypes derived from a human influenza virus and an HPAI virus would help bridge this gap of knowledge. In this report, we analyze the repertoire of reassortants between contemporary avian H5N1 and human H3N2 viruses by evaluating the phenotypes of 63 (2^6^-1) viral reassortants with HA and NA genes from an avian H5N1 virus and the six internal genes from either parental virus, assigned higher priority because only viruses with novel surface antigens may cause a pandemic. We used reverse genetics to derive the reassortant virus panel, and subsequently examined their replication characteristics in cell culture and their virulence in a mammalian system. Our *in vitro* and *in vivo* analyses revealed a high frequency of viable reassortants with a wide spectrum of virulence for mice, providing insight into their potential for future emergence in nature.

## Results

### Characterization of reverse genetics-derived H5N1 reassortant viruses in cell culture

**Figure 1:**
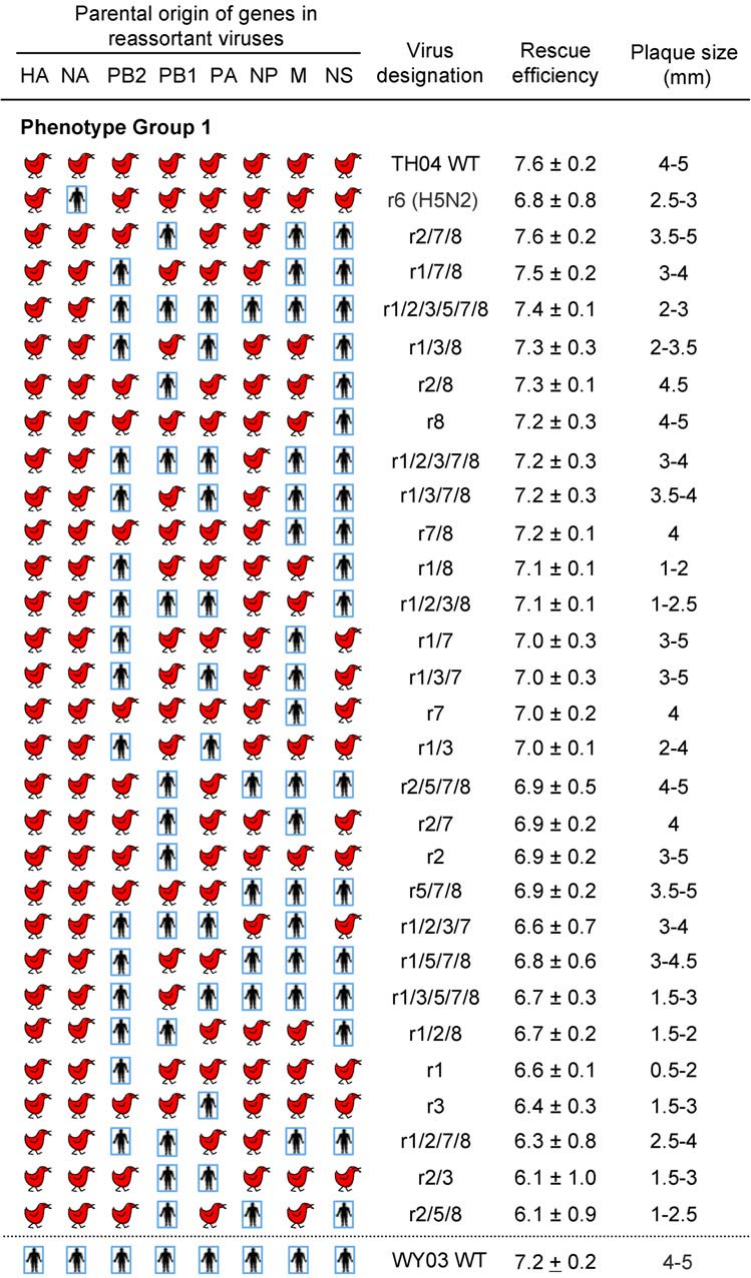
Characteristics of high yield avian-human reassortant viruses in cell culture. Human and chicken symbols denote the parental WY03 or TH04 source of each gene segment. The genotype of reassortants (r) is denoted by the genes derived from WY03 virus, designated by their segment number; 1:PB2. 2:PB1. 3:PA. 4:HA. 5:NP. 6:NA. 7:M. 8:NS. All others genes derived from TH04 do not bear a number. For example, r3/8 indicates the reassortant virus carries PA and NS genes from WY03 virus, and the remaining segments from TH04 virus. Rescue efficiency represents the virus titer (log_10_ pfu/ml) from cell cultures at 72 hours after transfection; geometric mean from 3 independent experiments.

**Figure 2:**
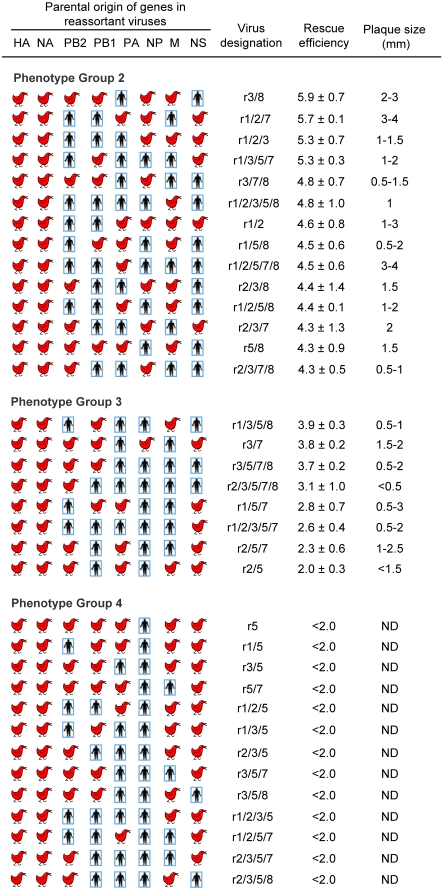
Characteristics of moderate to low yield avian-human reassortant viruses in cell culture. Symbols and virus nomenclatures are the same as described in Figure 1. Rescue efficiency represents virus titer (log_10_ pfu/ml) from cell cultures at 72 hours after transfection; geometric mean from 3 independent experiments. Plaque formation by reassortant viruses with <100 pfu/ml rescue efficiency was not determined (ND).

To generate the collection of human-avian reassortant viruses for this study, we first developed plasmid-based reverse genetics (rg) systems for the two parental viruses; A/Wyoming/3/2003 (subtype H3N2) (WY03) and A/Thailand/16/2004 (H5N1) (TH04) [17]. The parental WY03 virus showed α2,6 linked SA receptor specificity [18], replicated to high titers in MDCK cell culture, and was avirulent in mice (data not shown). The TH04 virus showed α2,3 receptor specificity [8], replicated efficiently in MDCK cells and was highly virulent for mice [17]. Virus recovery from plasmid DNA transfections was evaluated by quantitative plaque analysis at 72 hours (h) post-transfection; herein referred to as rescue efficiency. Cell cultures transfected with WY03 and TH04 rg plasmids yielded >10^7^ plaque-forming units (pfu)/ml of progeny virus, termed rgWY03 and rgTH04, which formed 4–5 mm diameter plaques, comparable to those of parental wildtype (wt) viruses (Figure 1). A wide range of virus yields and plaque diameters were obtained for each of the 63 H5N1 human-avian reassortant (rH5N1) plasmid transfections. In order to categorize the *in vitro* properties of each reassortant, rH5N1 genotypes were segregated into 4 phenotypic groups, according to their rescue efficiencies (Figures 1 and 2): **(1) rH5N1 genotypes with wt or near-wt replication efficiency**. Twenty-eight rH5N1 viruses (cell culture phenotype group 1) consistently yielded ≥10^6^ pfu/ml in the transfected cell cultures (Figure 1), which represented rescue efficiencies similar to those of rgWY03 and rgTH04. Most of the cell culture group 1 viruses formed ∼2–4 mm plaques in diameter (Figure 1). The efficient *in vitro* growth phenotypes of nearly one-half of the rH5N1 viruses in the group revealed a high frequency of functional compatibility between avian and human virus genes. **(2) rH5N1 genotypes with moderate cell culture replication impairment.** Fourteen rH5N1 viruses (22% of the rH5N1 genotypes) had rescue efficiencies between 10^4^ and 10^6^ pfu/ml (cell culture phenotype group 2), and most of these viruses formed 1–3 mm plaques (Figure 2). **(3) rH5N1 genotypes with severe cell culture replication impairment.** Eight reassortants (13% of the rH5N1 genotypes) yielded ∼10^2^–10^4^ pfu/ml from transfected cell cultures, with plaque size ranging from 0.5–2 mm (cell culture phenotype group 3 in Figure 2). **(4) Non-viable or marginally viable rH5N1 genotypes.** Thirteen rH5N1 viruses (∼20% of rH5N1 genotypes) yielded <100 pfu/ml from transfected cell cultures (cell culture phenotype group 4 in Figure 2). Their rescue efficiencies were 5 log_10_ pfu/ml lower than their rg parent viruses. The severe replication defects of viruses in this group may reflect structural or functional incompatibilities in avian-human viral RNA and/or protein complexes. Collectively, these categories guided our rationale for excluding reassortants with severe replication defects from further *in vivo* studies.

Notably, the severely impaired rH5N1 viruses in group 4 (Figure 2) were all characterized by the association of the nucleoprotein (NP) gene from WY03 virus with matrix (M) and/or nonstructural (NS) genes derived from TH04 virus. For example, the single gene reassortant r5 (group 4), which carried the NP from WY03 and the five other internal genes from TH04 had a rescue efficiency of <10^2^ pfu/ml. However, replacement of the TH04 NS gene with the WY03 NS in the same background increased rescue efficiency to ∼10^4^ pfu/ml (r5/8 virus, group 2, Figure 2), which was significantly higher than r5 (P≤0.0001). Further introduction of the WY03 M segment into this gene constellation restored the rescue efficiency and plaque size of the reassortant virus (r5/7/8, group 1, Figure 1) to nearly wt level (P≤0.0001). In contrast, introduction of polymerase complex genes did not improve replication (r5 replication is similar to r1/5, r2/5, r3/5, and r1/2/3/5; P>0.9) (Figure 2). Conversely, only 6 out of 28 rH5N1 viruses (group 1) that replicated efficiently had NP of human origin, in every case along with human NS (Figure 1). These observations suggest that the NP gene of WY03 origin preferentially interacts with M and NS genes of the same origin for optimal replication. In contrast, the NP gene of the TH04 avian virus appears to be more compatible with the M or NS of heterologous origin (e.g., r1/2/3/7/8 virus replication was similar to r1/2/3/7 or r1/2/3/8; P = 1.0). Although not all viruses with human NP and avian M or NS were severely impaired, they generally displayed significantly reduced replication, suggesting that avian M and/or NS may not be incorporated into seasonal human H3N2 viruses in the absence of avian NP.

Another remarkable gene incompatibility was noted with the r2/3/5/7/8 virus, bearing TH04 PB2 and the remaining five internal genes from WY03 virus (Figure 2, group 3). This reassortant virus formed tiny plaques (0.5 mm diameter) and had a very low rescue efficiency. This defect was repaired by providing a PA gene of avian virus origin; i.e., the rescue efficiency of r2/3/5/7/8 was significantly lower than r2/5/7/8 (Figure 1, group 1) (P<0.0001), suggesting a functional interaction of TH04 PB2 with the cognate avian PA gene. This finding suggests that reassortment of avian PB2 genes with human viruses may be linked to co-incorporation of the avian PA gene.

### Virulence of rH5N1 viruses in mice

**Figure 3:**
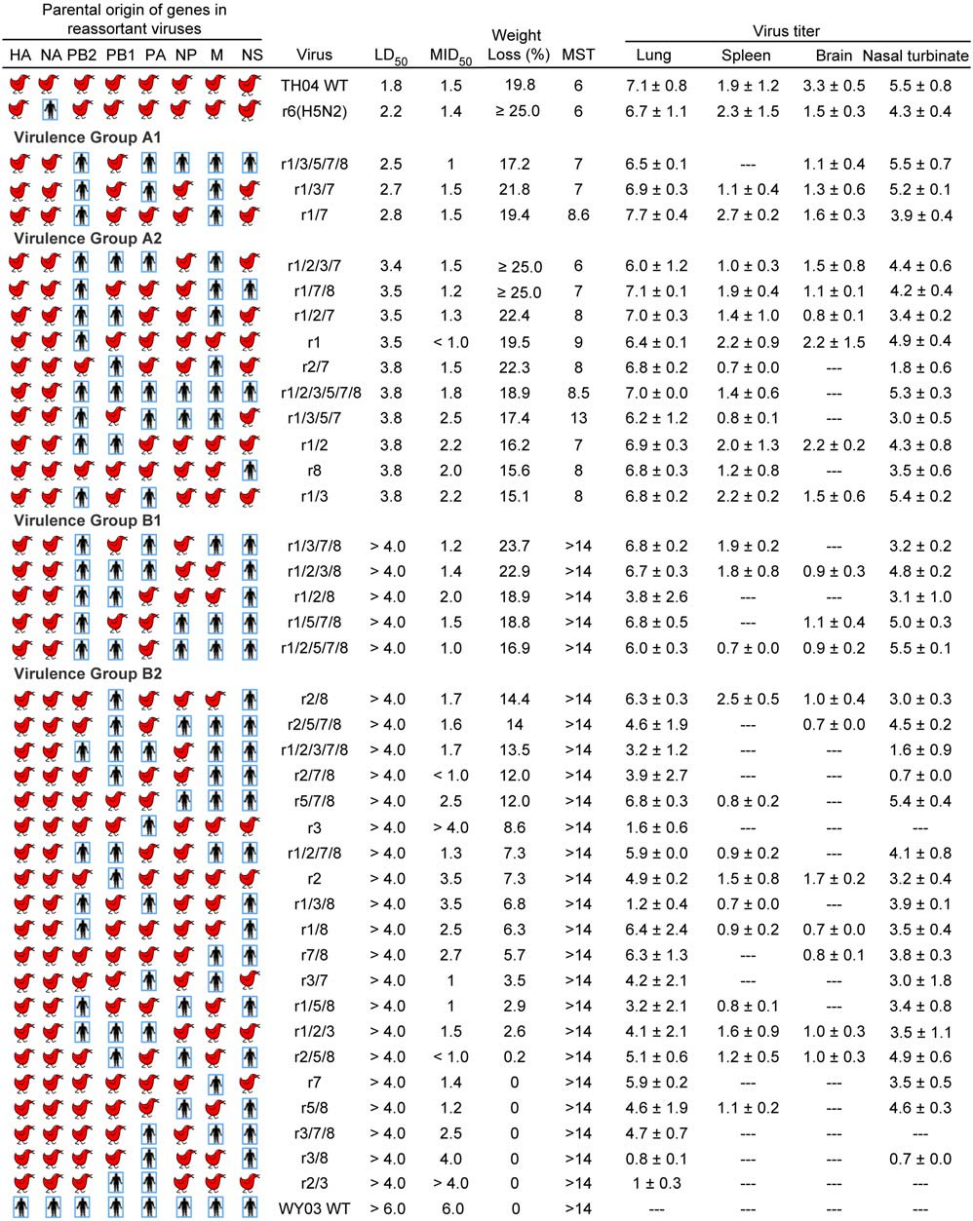
Replication of avian-human reassortant viruses in mice. Symbols and virus nomenclature are as in Figure 1. The mouse infectious dose (MID_50_) and lethal dose (LD_50_) are expressed as the log_10_ pfu required to give one MID_50_ or one LD_50._ Maximum mean weight loss was determined from five mice per group (percent weight loss relative to dpi 0) following intranasal infection with 10^4^ pfu. MST denotes the mean survival time in days following infection with 10^4^ pfu. Virus titer in lung, spleen, brain or nasal turbinate are geometric means of the log_10_ pfu at 4 dpi of three mice infected with 10^4^ pfu. LD_50_ values of rH5N1 in group A1 were significantly different from A2 and those from A1 and A2 were significantly different from TH04 WT (*P<*0.001) by analysis of variance. The — indicate that tissue titers were below limit of detection of the assay (0.7 log_10_ pfu/ml). Viruses are listed in ascending LD_50_ values. Viruses with identical LD_50_ are listed by descending weight loss.

A set of 38 rH5N1 viruses with cell culture replication efficiencies comparable to those of parental viruses, or with only modest reductions, were chosen for study in a BALB/c mouse model to assess their virulence in a mammalian host. The plasmid-derived rgTH04 virus was highly virulent for mice, as indicated by a very low intranasal 50% mouse infectious dose (MID_50_ = 10^1.5^ pfu) and lethal dose (LD_50_ = 10^1.8^ pfu) (Figure 3). This virus replicated to high titers (>10^7^ pfu/ml) in lungs by day 4 following an intranasal inoculation of 10^4^ pfu and caused >19% body weight loss. Viral replication was also detected at systemic sites, such as brain and spleen, recapitulating the outcome of infection with the wt TH04 isolate [17]. In contrast, replication of the rgWY03 virus in mice was very inefficient as evidenced by an MID_50_ of 10^6^ pfu and an LD_50_ of >10^6^ pfu (determination of MID_50_ for rgWY/03 required additional doses of 10^5^ and 10^6^ pfu to detect virus in tissues; data not shown). However, the reassortant virus bearing HA and NA from TH04 and the remaining genes from WY03 virus (r1/2/3/5/7/8) replicated efficiently (MID_50_ of 10^1.8^ pfu and titer of 10^7^ pfu/ml in the lung), suggesting that the HA and/or NA from WY03 lack appropriate interaction with receptors or other host factors in the mouse respiratory tract [19]. Most importantly, the internal genes from WY03 mediated efficient viral replication of r1/2/3/5/7/8 virus in mice validating the BALB/c mouse as a useful model to evaluate the influence of human/avian internal gene combinations on the virulence phenotypes of rH5N1 viruses.

Three rH5N1 viruses were highly virulent for mice, with an LD_50_<10^3^ pfu (Figure 3, group A1). Each of these rH5N1 had an MID_50_ of ≤10^1.5^ pfu, replicated to high titers in the lung (≥10^6.5^ pfu/ml), and caused >17% weight loss by 6–7 days post-infection (dpi) on average. The virulence of these viruses was comparable to that of wt TH04. In addition, the high frequency of virus detection in the spleen and brain of mice indicated systemic spread of these viruses, resembling infection with wt TH04. The ten viruses in group A2 were moderately virulent, with a mean LD_50_ value that was significantly different from that of the highly virulent group A1 viruses (*P*<0.001). The remaining 25 rH5N1 viruses in groups B1 and B2 exhibited low virulence phenotypes in mice with LD_50_ values >10^4^ pfu. However, five rH5N1viruses (Figure 3, virulence group B1) caused significant transient weight loss (>16%), clinical signs, such as ruffled haircoat and lethargy, and three viruses (r1/3/7/8, r1/2/3/8, r1/5/7/8) each caused mortality in a single mouse infected at 10^4^ pfu, suggesting potential for increased virulence at higher virus inoculums (data not shown). The other 20 rH5N1 viruses (Figure 3, virulence group B2) caused subclinical infections in mice, with minor weight loss (<15%). These viruses spread to the spleen and /or brain sporadically and their pulmonary replication capacity ranged from substantially efficient to nil. Although many rH5N1 viruses with high rescue efficiencies and large plaque phenotypes also displayed highly virulent phenotypes in mice, several rH5N1 viruses belonging to virulence group B2 (i.e., r2/7/8 and r1/3/8) had high rescue efficiencies but did not replicate well in mice. This finding highlights the limitations of inferring *in vivo* virulence properties solely from efficient *in vitro* replication characteristics.

Interestingly, r1/3/5/7/8, one of the most virulent rH5N1 among the 38 reassortants inoculated into mice had a gene constellation resembling that of the pandemic viruses from 1957 and 1968. In 1957, HA, NA and PB1 genes from an avian H2N2 virus were introduced into the circulating human H1N1 virus and caused the so-called “Asian flu” pandemic, whereas the 1968 “Hong Kong” pandemic virus incorporated the HA and PB1 genes from an avian donor [3]. In this study, a virus carrying HA, NA and PB1 of avian origin and the remaining genes from a human virus, namely r1/3/5/7/8, was highly virulent for mice (LD_50_ = 10^2.5^ pfu). In contrast, a reassortant virus (r1/2/3/5/7/8) with all the internal genes from WY03 virus, including PB1, caused minimal mortality and had a significantly different LD_50_ (1.3 log_10_ pfu increase; *P*<0.001), suggesting that the PB1 of contemporary H5N1 viruses can reassort into circulating H3N2 viruses and increase their virulence for mice.

Efficient viral replication at the lower temperature of the upper respiratory tract is thought to be essential for droplet transmission of influenza virus between humans. Avian influenza viruses with a PB2 polymerase bearing glutamic acid at position 627 instead of lysine have decreased replication at 33°C in mammalian cells [20],[21],[22]. Although both WY03 and TH04 viruses have lysine at position 627 in PB2, it is not known whether new avian and human gene constellations would compromise viral replication at lower temperature. To address this question, we determined reassortant viral titers in the nasal turbinates collected at 4 dpi. We found that in general, rH5N1 viruses replicated less efficiently in nasal turbinates than in lungs, as reported previously [21]. Interestingly, some reassortants (i.e., r2/7, r2/7/8, and r3/7/8) showed extremely low replication in nasal turbinates despite considerable titers in lungs (Figure 3). These reassortants would be expected to lack efficient transmissibility by generation of nasal secretion droplets.

### Replication of rH5N1 viruses in differentiated primary human tracheobronchial epithelial (HTBE) cells

**Figure 4:**
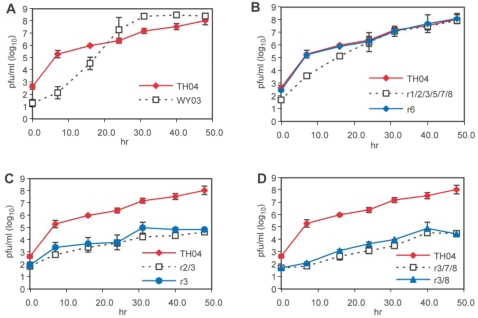
Replication kinetics of avian-human reassortant viruses in differentiated human tracheobronchial epithelial (HTBE) cells. HTBE cells were infected in duplicate with parental TH04 and WY03 (A) or rH5N1 viruses (B, C, D) at an moi of 0.02; progeny viruses were collected and titrated on MDCK cells.

Although mice are regarded as a useful mammalian model for studying the replication of HPAI viruses, the species differences between humans and mice mandate studies with models from the target species to complement the data. The epithelial cells of the respiratory tract are the primary targets of influenza infection. Therefore, we used *in vitro* differentiated HTBE cultures to evaluate the replication potential of the rH5N1 viruses in humans [23]. HTBE cells were infected with rgWY03 and rgTH04 viruses, or each of 38 rH5N1 viruses that were previously analyzed for virulence in mice. We quantified virus progeny released into the apical side of the pseudostratified epithelium because budding of HPAI H5N1 virus in the HTBE model remains polarized (data now shown). As shown in Figure 4A, both rgTH04 and rgWY03 parental viruses replicated efficiently in the HTBE cells. The rapid rise of WY03 virus titers to 10^8^ pfu/ml at 32 h post-infection was consistent with the efficient spread of human viruses in HTBE cells, as described previously [23]. The plateau in WY03 virus production may be caused by virus-induced cell death, first noted at 40 h post-infection. In contrast, HTBE cells infected with rgTH04 virus showed no cytopathology and virus progeny increased steadily throughout the 56 h infection. The majority of rH5N1 viruses produced ≥10^4^ pfu/ml by 24 h post-infection, and the growth kinetics were similar to parental rgTH04 or slightly delayed (e.g., r1/2/3/5/7/8 in Figure 4B and data not shown for others). In comparison, four rH5N1 viruses, r/3, r2/3, r3/8, r3/7/8, replicated substantially less efficiently in the HTBE cells (Figure 4, C and D). Interestingly, these four viruses also replicated poorly in mice; had MID_50_ values of ≥4 log_10_ pfu and caused minimal weight loss (Figure 3). These results supported the virulence data provided by the mouse model.

### In vitro viral polymerase activity

**Figure 5:**
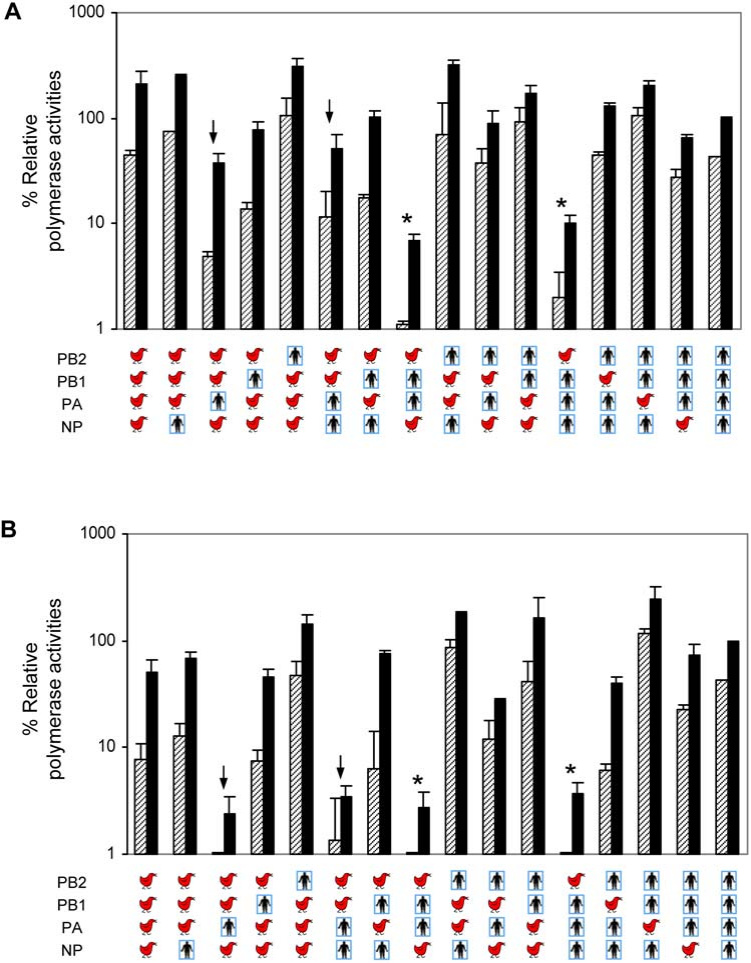
Polymerase activity of avian-human viral ribonucleoprotein (RNP) complexes. (A) A549 cells were transfected in duplicate with pPol1-NS-Renilla and pSV40-Luc reporter plasmids, together with plasmids expressing PB2, PB1, PA and NP from either WY03 (human symbol) or TH04 (chicken symbol) viruses. Cells were incubated at 33°C (hatched bars) or 37°C (solid bars) for 24 hours and cell lysates were analyzed to measure Renilla and firefly luciferase activities. The latter was used to normalize transfection efficiency. Values shown represent the activities of each RNP relative to that of WY03 measured at 37°C (100%). (B) Viral RNP activities derived from WY03 (human symbol) or VN04 (chicken symbol) viruses are shown as described in panel A.

To study the mechanisms underlying the differences in the replication phenotypes of certain rH5N1, we exploited a mini-genome reporter assay which dissects the function of the viral ribonucleoprotein (RNP) complex from the rest of the viral gene products [24],[25]. The 16 possible RNP combinations of PB2, PB1, PA and NP from either the TH04 or WY03 viruses were studied at 33°C or 37°C, to recapitulate the temperatures of the upper and lower respiratory tract, as reported previously [20]. Another panel of RNP combinations with A/Vietnam/1203/2004 (VN04) viral genes replacing TH04 genes was also analyzed in parallel to extend the results for other H5N1 viruses. The human RNP was almost equally active at 33°C and 37°C, whereas the avian RNP activity was substantially reduced at 33°C despite the presence of lysine at position 627 of PB2, in both TH04 and VN04 backgrounds (Figure 5). The RNP constituted by PB1 and PA from WY03 virus and PB2 from TH04 (or VN04) virus resulted in extremely low polymerase activities at 33°C and 37°C (Figure 5A and B, RNP denoted by asterisks). Although the RNP complexes carrying PB2 and PB1 from TH04 and PA from WY03 virus showed partially reduced polymerase activity, a similar combination derived from VN04 and WY03 viruses showed a more pronounced loss of replication activity (Figure 5A and B, RNP denoted by arrows). The reduced polymerase activities of these gene constellations were consistent with the low viral titer from lungs and nasal turbinates of mice infected with reassortant viruses r3/7/8, r3/8, r3 and r2/3 (Figure 3). Interestingly, the polymerase activity of the RNP with PB1 from TH04 and the other proteins from WY03 was comparable to that of the wt WY03 RNP. These findings indicated that the increased mouse virulence attributed to avian PB1 in the WY03 genetic background (r1/3/5/7/8) may not directly result from stimulation of the polymerase activities of the RNP. Alternative hypotheses to reconcile these observations would include an *in vivo* role for PB1 in RNP function, a possible modulation of host cell function by PB1-F2, or unknown interactions of PB1 with the remaining 4 genes absent in this assay: HA, NA, M and NS.

### Characterization of a reverse genetics-derived H5N2 reassortant virus

Although H5N2 subtype viruses have been isolated from poultry in North America and Asia for many years [26], it is not clear whether the N2 derived from contemporary human H3N2 virus can support the efficient replication of a reassortant virus bearing the HA from circulating H5N1 virus. Balanced HA and NA activities are critical for efficient influenza virus infection and replication in various hosts. The HA of contemporary H5N1 viruses has retained a strong preference for α2,3 linked SA [7],[8]. In contrast, the NA derived from H3N2 human seasonal isolates has adapted over time to acquire α2,6 SA specificity [27],[28]. To evaluate the H5N2 reassortant, the NA of TH04 was replaced with the N2 from WY03 virus by reverse genetics. This virus, termed r6 (H5N2), was virtually identical to wt TH04 in rescue efficiency and replication in HTBE cells (Figure 1 and Figure 4B). In addition, this reassortant was highly virulent in mice, with an LD_50_ comparable to the parental TH04 virus (Figure 3). These results suggested that the NA activity from circulating H3N2 viruses can functionally support the activity of the H5 HA and promote H5N2 virus spread in the mammalian host.

## Discussion

The influenza pandemics of the past century were caused by viruses carrying at least one internal gene of avian origin and a novel HA subtype that acquired α2,6 SA receptor binding specificity [3]. While many studies have focused on adaptive mutations in the avian HAs required for acquisition of human receptor specificity, little is known about the importance of the avian virus internal genes in pandemic emergence [7],[29],[30]. In this report, we used reverse genetics to systematically study reassortants with each of the 63 possible combinations of internal genes from contemporary avian and human viruses; all with H5N1 surface protein genes.

Collectively, these studies revealed that certain genes, such as human NP and avian PB2, often caused severe replicative impairment in cell culture when transferred alone to the heterologous virus background, whereas transfer of other genes, such as PB1, was less detrimental. The incompatibility of the human NP with a full complement of avian influenza genes was noted in early studies with Fowl Plague virus [13]. This is significant because the NP gene of influenza virus plays an important role in host range specificity [5],[31],[32]. In this report, we provide evidence suggesting that reassortants with NP of avian origin in a human virus background can replicate efficiently in mammalian cell cultures. This phenotype does not require the presence of other avian virus internal genes, whereas the NP gene of human origin depends on cognate NS and M genes for expression of the efficient replication phenotype. The best characterized event of the viral infectious cycle involving NP, M, and NS gene products is the nuclear export of viral RNP. In the nucleus, the influenza nuclear export protein (NEP; encoded by the NS gene) interacts with the M1 protein, which binds to the newly assembled viral RNP. NEP also interacts with host protein CRM1, thereby directing the nuclear export of the viral RNP complex [33],[34],[35]. Although a direct interaction between NEP and NP proteins has not been shown, the severely defective growth of reassortants possessing heterologous M and NS relative to NP suggests an unidentified crosstalk between these viral proteins, with the possible involvement of a host protein(s).

Striking viral phenotypes were also evident in rH5N1 viruses with heterologous polymerase subunits. The PB1 protein interacts with PA and PB2 forming transcriptionally active heterotrimers [36],[37],[38]. Although a direct interaction between PB2 and PA has never been reported, our genetic analyses pointed towards a specific interdependence between PB2 and cognate PA genes of avian origin, either through direct protein-protein interaction or concerted interaction with other viral or host factor(s). Interestingly, natural avian-mammalian reassortant viruses isolated from humans and swine possess PB2 and PA of the same host origin and sometimes carry a PB1 derived from a virus adapted to a third host species [39],[40]. Thus, linkage between avian PB2 and PA would be expected in the event of reassortment between an H5N1 virus and a seasonal H3N2 virus from humans.

The role of the avian PB1 genes in the emergence of reassortant viruses that caused the 1957 and 1968 influenza pandemics has remained enigmatic. This study shows that incorporation of an avian PB1 gene into a background of human virus internal genes significantly increased mouse virulence. We postulate that acquisition of the avian PB1 gene, as was seen in the 1957 and 1968 pandemic influenza strains may be a critical factor in the early stages of a pandemic, allowing the emerging reassortant to overcome competition with seasonal influenza viruses by enhancing its replication or virulence. Our results, therefore, have implications for assessing the potential virulence of novel reassortant viruses possessing human virus internal genes and PB1 from currently circulating H5N1 viruses.

Although reassortment between two different viruses could yield 254 possible new genotypes, this study characterized the subset of 63 genotypes with H5N1 surface antigens, of highest public health significance. In addition, these studies show that a reassortant virus with NA from a contemporary human H3N2 virus and the remaining 7 genes from TH04 replicated efficiently and was as lethal as wt H5N1 virus in mice, indicating that the current human N2 is compatible with the receptor binding function of the H5 HA. Although we did not analyze all the 63 additional genotypes carrying H5N2 surface genes, we anticipate that their virulence would be similar to their rH5N1 counterparts. However, these data should be interpreted in a broader context of human and avian influenza virus replication and evolution. The genotype of the rH5N1 that would emerge from natural co-infection is dictated by many factors besides the replication competency of a given reassortant. Dual infection of a single cell with human and avian influenza viruses involves co-replication of two genomes that may complement, interfere, and compete with each other. These events and the subsequent expansion of the reassortan will be further conditioned by the host species and tissue tropisms of the parental viruses and resulting reassortants. Ultimately, while use of reverse genetics technology to generate reassortants provides an experimental platform free of these many variables, natural reassortment between two viral genomes, and the consequences therein, are more complex.

In summary, we report a strikingly high level of compatibility between avian and human virus genes. Because few studies have described naturally occurring or experimentally derived avian-human reassortants, our results were surprising in that almost half of the rH5N1 viruses tested showed a high frequency of functional compatibility between avian and human virus genes. In addition, approximately 1 in 5 of all possible H5N1 reassortants was lethal for mice at doses below 10^4^ pfu. The highly virulent reassortant genotypes identified in this study suggest that introduction of certain H5N1 viral segments into circulating human H3N2 viruses may increase their virulence for mice and perhaps other mammalian species. In addition, the moderately virulent reassortant viruses could circulate in a mammalian host, evolve by compensatory and/or adaptive mutations, and become more virulent for humans. The results of this study, therefore, underscore the necessity for enhanced viral surveillance strategies, which monitor reassortment events in nature to reduce the public health threat posed by H5N1 HPAI viruses currently circulating in three continents.

## Materials and Methods

### Viruses and cells

A/Thailand/16/2004 (TH04) and A/Vietnam/1203/2004 (VN04) H5N1 viruses and A/Wyoming/3/2003 (WY03) H3N2 virus obtained from the WHO Global Influenza Surveillance Network were provided by Alexander Klimov (CDC, Atlanta, USA). Madin-Darby canine kidney (MDCK) and human lung carcinoma (A549) cells were obtained from the American Type Culture Collection and propagated in Dulbecco's Modification of Eagle's Medium with 10% fetal bovine serum. Viral infectivity was determined by plaque assay on MDCK cells as described [41]. Reassortant viruses containing any segment derived from the H5N1 virus were generated in compliance with the Institutional Biosafety Committee and NIH Guidelines for Research Involving Recombinant DNA Molecules. Viruses were handled in biosafety level 3 containment, including enhancements required by the U.S. Department of Agriculture and the Select Agents program http://www.cdc.gov/od/ohs/biosfty/bmbl5/bmbl5toc.htm.

### Generation of reassortant viruses by reverse genetics

RT-PCR amplicons of the eight viral genes from WY03 and TH04 viruses were cloned into a dual-promoter plasmid for influenza A reverse genetics [42]. Virus rescue was performed by plasmid DNA transfection into co-cultured 293T/MDCK cells [42]. Culture medium from the transfected cells was harvested at 72 h and analyzed by plaque assay on MDCK monolayers. The plaque count and diameter were recorded as a measure of the virus rescue efficiency from plasmid DNA. DNA transfection of each genotype was performed at least three times independently. WY03 and TH04 rg plasmid sets were included as controls during each reassortant rescue to evaluate experimental variation. Viruses with H5 HA were propagated in 10–11 days old embryonated chicken eggs. The H3N2 virus was propagated in MDCK cells in the presence of 1 μg/ml TPCK-treated trypsin. Following propagation, the full genomes of reassortant viruses were sequenced to confirm presence of parental virus sequence.

### Luciferase mini-genome reporter assay

A549 cells cultured in 24-well tissue culture plates were co-transfected with pPol1-NS-Renilla (100 ng) encoding a reporter mini-genome under transcriptional control of the human RNA polymerase I, pSV-Luc (200 ng) encoding firefly luciferase under SV40 virus RNA polymerase II promoter control, and four plasmids expressing viral PB2, PB1, PA, NP (50 ng each) from the strain of interest. Twenty-four hours after the transfection, the cell lysates were harvested and further diluted to perform the dual luciferase assay according to the manufacturer's protocol (Promega). The influenza polymerase catalytic activity derived from the Renilla luciferase plasmid (pPol1-NS-Renilla) was corrected to account for well-to-well differences in transfection efficiency using the firefly luciferase activity values from pSV-Luc plasmid.

### Pathogenicity studies in mice

Groups of 6-8 week old female BALB/c mice (Jackson Laboratories, Bar Harbor, ME) were placed under light anesthesia and inoculated intranasally with 50 μl of serial 10-fold dilutions of infectious virus in PBS. For reassortant viruses tested, 10^4^ pfu of virus was the highest dose used to infect mice; for WY03 virus, 10^6^ pfu of virus was tested. Three mice from each group were euthanized at 4 days post-infection (dpi) and nasal turbinates, lungs, spleens, and brains were harvested, immediately frozen on dry ice, and stored at −80°C until further processing. Whole tissues were thawed, homogenized in 1 ml of cold PBS, and clarified by centrifugation (2,200×*g*) at 4°C. Virus titers of homogenates were determined by plaque assay in MDCK cells. Five additional mice in each group were monitored daily for clinical signs for 14 dpi. Mice that lost more than 25% of their body weight were euthanized humanely. The fifty percent mouse infectious dose (MID_50_) and fifty percent lethal dose (LD_50_) were calculated and expressed as the pfu value corresponding to 1 MID_50_ or LD_50_. Animal studies were conducted per approved Institutional Animal Care and Use Committee protocols.

### Statistical analysis

Statistically significant differences of rescue efficiencies of avian-human reassortants in cell culture were determined by F-test adjusted for multiple comparisons. LD_50_ and MID_50_ values were calculated using the method of Reed and Muench [43]. Statistically significant differences between LD_50_ values of viruses in virulence group A1 and A2 were determined by comparing groups A1 and A2 to TH04 WT and group A1 to group A2 using an analysis of variance performed by an F test for multiple comparisons.

### Viral replication in differentiated primary human tracheobronchial epithelial cells

Growth and differentiation of primary human tracheobronchial epithelial cells were performed as described previously [23],[30],[44]. Briefly, primary cells (passage level 3) were seeded in porous membrane inserts (Corning, 4.5 μm, 12 mm diameter) at the density of 5×10^4^ cell/cm^2^. Three days after seeding the cells, the medium from the apical side was removed and the confluent monolayers were cultured at an air-liquid interface. The medium from the basal compartment was replaced daily, and the *in vitro* differentiation of primary cells was achieved after 4–6 weeks. Differentiated cells with trans-epithelial electrical resistance of ≥300Ω cm^2^ were used in our study. Kinetic analysis of reassortant virus growth was performed after infection of the monolayer at a multiplicity of infection (moi) of 0.02 pfu/cell as described [23],[30]; apically released virus was harvested at the appropriate times and analyzed by plaque assay.

### Accession numbers

The GenBank (http://www.ncbi.nlm.nih.gov/sites/entrez) accession numbers for the genes described in this paper are: EU268216 (A/Thailand/16/2004, PB2 gene), EU268217 (A/Thailand/16/2004, PB1 gene), EU268218 (A/Thailand/16/2004, PA gene), EU268219 (A/Thailand/16/2004, HA gene), EU268220 (A/Thailand/16/2004, NP gene), EU268221 (A/Thailand/16/2004, NA gene), EU268222 (A/Thailand/16/2004, M gene), EU268223 (A/Thailand/16/2004, NS gene), EU268224 (A/Wyoming/03/2003, PB2 gene), EU268225 (A/Wyoming/03/2003, PB1 gene), EU268226 (A/Wyoming/03/2003, PA gene), EU268227 (A/Wyoming/03/2003, HA gene), EU268228 (A/Wyoming/03/2003, NP gene), EU268229 (A/Wyoming/03/2003, NA gene), EU268230 (A/Wyoming/03/2003, M gene), EU268231 (A/Wyoming/03/2003, NS gene).
